# An introduction to agent‐based models as an accessible surrogate to field‐based research and teaching

**DOI:** 10.1002/ece3.6848

**Published:** 2020-10-02

**Authors:** Kilian J. Murphy, Simone Ciuti, Adam Kane

**Affiliations:** ^1^ School of Biology and Environmental Science and the Earth Institute University College Dublin Dublin Ireland

**Keywords:** accessible resource, agent‐based model, computational tools, ecology, fieldwork, inclusive resource, NetLogo, open‐source resource, quantitative methods

## Abstract

There are many barriers to fieldwork including cost, time, and physical ability. Unfortunately, these barriers disproportionately affect minority communities and create a disparity in access to fieldwork in the natural sciences. Travel restrictions, concerns about our carbon footprint, and the global lockdown have extended this barrier to fieldwork across the community and led to increased anxiety about gaps in productivity, especially among graduate students and early‐career researchers. In this paper, we discuss agent‐based modeling as an open‐source, accessible, and inclusive resource to substitute for lost fieldwork during COVID‐19 and for future scenarios of travel restrictions such as climate change and economic downturn. We describe the benefits of Agent‐Based models as a teaching and training resource for students across education levels. We discuss how and why educators and research scientists can implement them with examples from the literature on how agent‐based models can be applied broadly across life science research. We aim to amplify awareness and adoption of this technique to broaden the diversity and size of the agent‐based modeling community in ecology and evolutionary research. Finally, we discuss the challenges facing agent‐based modeling and discuss how quantitative ecology can work in tandem with traditional field ecology to improve both methods.

## Graphical abstract

The COVID‐19 global lockdown may be an early harbinger for future disruptions to fieldwork under climate change scenarios. Agent‐Based models are a powerful and accessible way to surrogate lost field seasons to continue these projects or begin new ones. This innovative method is improving constantly and is an excellent tool for teaching and research in natural science.

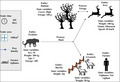

## 1 INTRODUCTION

Fieldwork encompasses any practical work taking place outside the laboratory for data collection and learning (Lock, 1998). Field data collection is essential to investigate long‐term ecological processes and observe new phenomena for the first time. Moreover, university fieldwork is a central component of coursework as fieldwork‐based skills including project design, surveying, data curation, and risk assessment are vital for students seeking work in a competitive job market (Pool & Sewell, 2007). Thus, fieldwork for research and teaching purposes provides essential training and experience for early‐career researchers (Peacock & Bacon, 2018).

Despite these benefits, fieldwork is an expensive endeavor that is not accessible to all (Giles et al., 2020). Access to resources and the privilege of delaying other responsibilities to conduct fieldwork create a barrier to skills that disproportionately affect minorities and the disabled community (Giles et al., 2020; Healey et al., 2002). The opportunity to work in remote areas on exotic species is thus afforded to a select few in the community. The COVID‐19 global pandemic has extended these barriers across the community and disrupted projects throughout the world leading to the loss of field seasons for many researchers due to lockdowns and travel restrictions (Fikrig, 2020). For early‐career researchers and students, this has led to amplified stress and anxiety regarding the uncertain future of their projects (Kimbrough, 2020; Leigh Hester, 2020; Tercel, 2020). Future scenarios such as economic downturn and climate change may also present disruptions to research and teaching in life sciences (Cagnacci et al., 2012). Adapting to “the new normal” presents an opportunity to integrate computational tools and quantitative methods into life sciences learning and research.

Throughout this paper, we will discuss agent‐based modelling (also known as Individual‐Based Modeling but hereafter referred to as agent‐based modelling) as an accessible and powerful tool with broad applications in the field of ecology and evolution. Our aim was to describe the many applications of agent‐based modelling for teaching and research in life sciences as a powerful computational surrogate method when field research is not possible.

## 2 WHAT IS AN AGENT‐BASED MODEL?

Agent‐based models (ABMs) are a simulation tool capable of testing and teaching biological theory from the microbe level to whole ecosystems (McLane et al., 2011). It is a tool particularly suited to ecology as it is composed of individual agents and an environment. Agents populate a spatial environment and interact with this environment as well as with each other, giving it an advantage over most traditional modeling tools (Huston et al., 1998). Over the past twenty‐five years, ABMs have been used across a range of projects to study wildlife movement, behavior, and management among others (Benadi & Gegear, 2018; Bryson et al., 2007; Hartig et al., 2014; Tang & Bennett, 2010). Simulations allow for dynamic studies of ecological relationships. A central facet of ecological studies is understanding these relationships and informing practical management decisions from empirical science (Margules & Pressey, 2000). The software used in ABMs is continually developing in line with technological advancements. The integration of adaptation, learning, fuzzy logic, randomness, and evolution into ABM is now being used to examine how systems emerge and how their influence over the environment and its inhabitants change over time (DeAngelis & Diaz, 2019).

ABM simulations offer insight into the adaptation and function of systems over time and space when access to field data is dangerous, unavailable, or simply impossible to collect (Cagnacci et al., 2012; Sokolowski & Banks, 2009). As COVID‐19 creates a major barrier for fieldwork in the summer of 2020, ABMs offer an alternative method to collect data and test hypotheses in a collaborative and inclusive way.

### 2.1 Model composition

**FIGURE 1:**
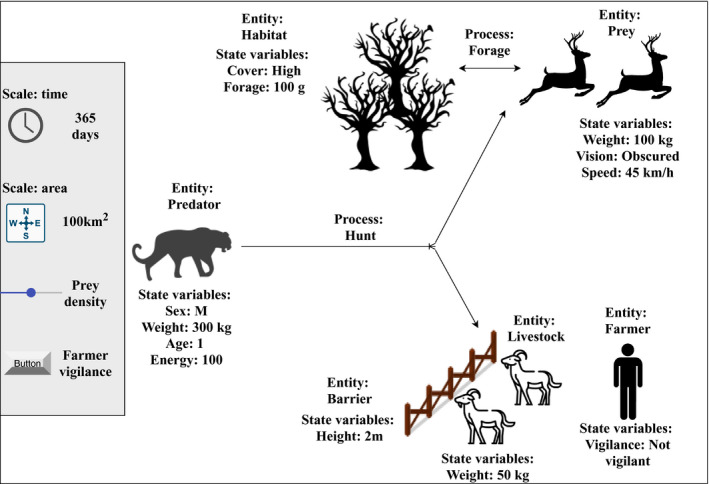
An example of an agent‐based model design for predator‐prey dynamics in an agricultural landscape. The predator's objective is to catch prey and the outcome of that is dependent on its state variables, for example, sex and age may dictate hunting range. The prey animals' objective is to forage and survive. To achieve this, they must enter certain parts of the environment which may increase their chance of encountering a predator. The farmer's objective is to keep the livestock alive, whether a predator has interacted with the farm previously can affect their “vigilance”, thus if livestock is undefended it may be more accessible to the predator. In this example, each agent's objective affects other agents by altering the environment and the state variables which drive decision making. In the grey section (left) is the spatiotemporal scale of the model and the developer variables, these variables can influence the model during an experiment or prior to one, for example, changing prey accessibility via prey density and farmer vigilance

ABMs are composed of data‐fed agents and an environment. An agent is an entity with a set of parameters and a defined objective that interacts with other entities within the model to achieve this objective. In less abstract terms, this could be a model predator looking for prey. The environment is the world the agents occupy and is often represented in a patchwork. Each patch has parameters which affect how the agents interact with it, for example, a grassland environment where some patches are occupied by prey animals. Through these interactions, parameters for both agents and the environment can change; thus, future interactions are stochastic and allow for the emergence of novel behavioral and landscape patterns to emerge, for example, emergence of home range in areas of food. ABMs can be spatially and temporally explicit. The spatial and temporal scales are both defined by the user—a crucial step that is dependent on the objectives of the study. See Figure 1 for a graphical representation of an example agent‐based model aimed at examining predator–prey interactions in a mixed‐agricultural environment.

The parameters (e.g., speed of the predator, density, and detection distance of prey) are important as model results are sensitive to these conditions (Saadat et al., 2018). There are techniques available to choose these initial parameters such as using observations from field sites, existing datasets, or publications. Similarly, there are a variety of statistical methods for tuning initial model parameters for example sensitivity analysis, microsimulation, and machine learning (Calvez & Hutzler, 2006; Hassan et al., 2010). Input‐data in ABMs are data that influence processes within the model environment but are not in turn influenced by the simulation; for example, daily precipitation input‐data effects on simulated soil moisture (Eisinger & Wiegand, 2008). For this reason, these data are differentiated from entity variables and initial model parameters (Grimm et al., 2010). These data may be selected from field sites such as weather stations, previous publications, or generated using statistical modeling.

### 2.2 The ODD protocol

**FIGURE 2:**
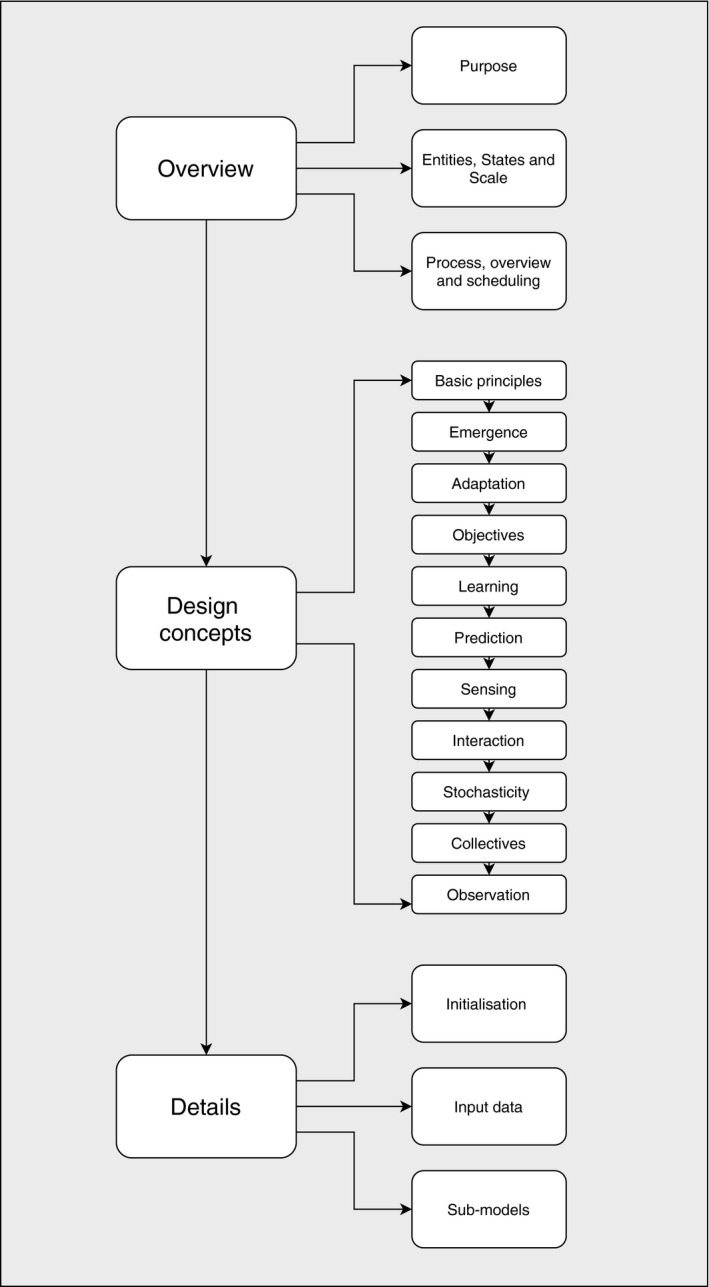
Structure of the ODD protocol which should inform model development. The categories O (Overview), D (Design concepts), and D (Details) are meant as comments but are not used in ODD model descriptions. The sub‐headings are used to describe the model thoroughly from concept to mechanical functionality

Early scepticism of this tool has led to marked improvements in standards and protocols such that a framework now exists to ensure repeatability and consistency across agent‐based models (Hare & Deadman, 2004; Grimm et al., 2010). The result of this development is that ecologists can simulate testable hypotheses in realistic environments with complex dynamics between agents for both hypothetical and real‐world scenarios without having to travel for fieldwork. This framework known as the Overview, Design Concepts, and Details protocol (ODD) was developed by Grimm et al. (2006). This is a standardized communication method to ensure that model development is understandable and repeatable by the scientific community, which is a staple of good research. The ODD protocol is a structured report for presenting the rationale, evidence and supporting information of the model development process (Figure 2). It entails the following:

1. Overview: General information and context of the model
2. Design concepts: Strategic considerations and internal methods
3. Details: Technical methodology and details of their use in the model.

Widely used since its conception, the ODD protocol ensures the high standards of ecological research are adhered to during model development (Grimm et al., 2010; Grimm et al., 2020). It ensures the research question, no matter how complex, is transparent to scientific review such that the theoretical foundations of the model are robust and the formulation of model features is rigorous. The criteria listed under the ODD protocol dictates that each entity is fitted with a complex adaptive system (CAS). Implementation of these criteria creates structured entities that provide greater value in basic or advanced models. The framework ensures that theoretical foundations are included computationally and prevents ad hoc programming so that the model is a realistic system for applied research (Railsback, 2001). See Polhill, Parker, Brown, & Grimm (2008) for examples of three ABM's described via the ODD protocol.

### 2.3 Software

**FIGURE 3:**
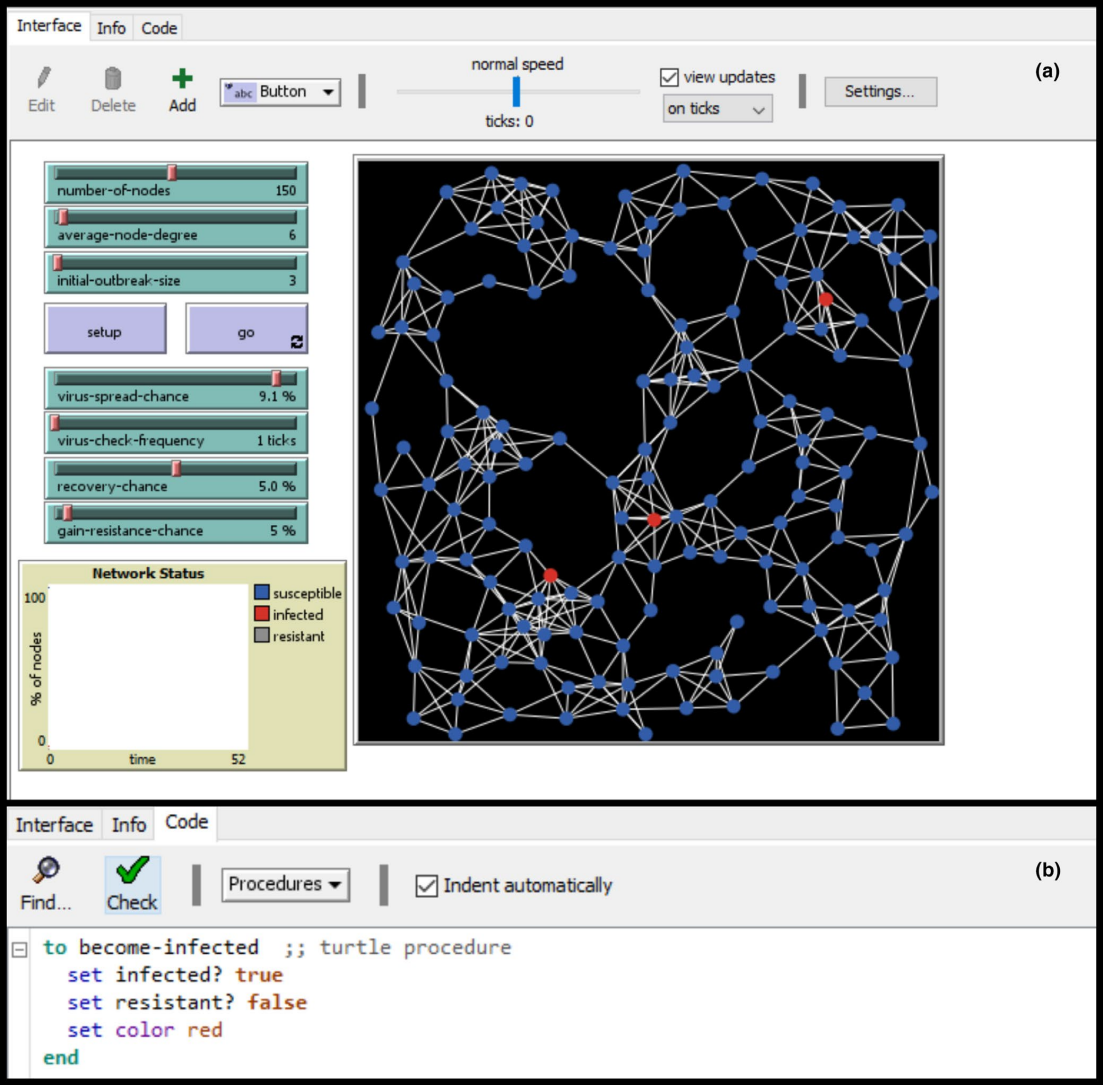
Example of an agent‐based model in the NetLogo environment (version 6.1.1). This software is recommended due to its understandable and easy to use graphical user interface (a) and its simple programming language (b) that does not require advanced programming skills to create models. This model is an ABM SIR model that demonstrates the spread of a virus (red cells) through a network of uninfected individuals (blue cells). It is open‐source and available in the Netlogo model library (Stonedahl & Wilensky, 2008)

Much of the software to create and run ABMs is open‐source and hosts an inclusive community that is essential to newcomers to the field. Innovation in ABMs is fueled by the collaborative and diverse community that extends this tool to simulate increasingly complex phenomena (Heckbert, Baynes, & Reeson, 2010). The availability of software packages and the computational power required to develop a model has greatly improved in recent years facilitating greater accessibility across disciplines (Railsback et al., 2006; Salecker et al., 2019; Thiele, 2014). Table 1 displays the range of software available for developing an ABM. We recommend ecologists to use NetLogo as it is an open‐source and free‐to‐use platform. NetLogo also boasts simple programming language, graphical user interface, and a comprehensive library of resources comprising community models, code documentation, and cloud services (Figure 3). The method's accessibility has promoted the growth of a diverse community and research output. The remainder of this paper will focus on the use of NetLogo for the development of ABMs for teaching and research, see Abar et al. (2017) for a comprehensive review of ABM software if interested in other software.

**TABLE 1:** Toolbox of products to develop agent‐based models recommended for ecologists

Platform	Developer	Programming language	Operating system
AnyLogic	The AnyLogic Company; Oakbrook Terrace, Illinois, USA	Java	Microsoft Windows 7 and after; SP1, x64; Apple Mac OS X 10.10 Universal; SuSE Linux, x64 (with installed GTK+
Cougaar	Cougaar Software Inc.; Vienna, Virginia, USA]	Java	Windows 98; Windows NT; Windows XP; Linux; Mac OS X; and Java‐1.4‐capable PDA
Framsticks	Poznan University of Technology, Poznan, Poland	FramScript	Windows; Linux; Mac OS X
MASON	George Mason University, Fairfax, Virginia, USA	JAVA	Any Java supporting machine (version 1.3 or higher)
NetLogo	Northwestern University, Evanston, Illinois, USA	NetLogo	Any Java supporting machine (version 6 or higher)
SARL	Stéphane Galland, Burgundy Franche‐Comté University, France; Nicolas Gaud, Burgundy Franche‐Comté University, France, Sebastian Rodriguez, Advanced Informatics Technology Research Group, Tucuman, Argentina	SARL/Java	Any Java supporting machine (version 1.8 or higher)
Starlogo	Mitchel Resnick, Eric Klopfer, and others at MIT Media Lab and The MIT Scheller Teacher Education Program, Massachusetts Institute of Technology; Cambridge, MA, USA	StarLogo (an extension of Logo)	Mac OS X v10.2.6 or higher with Java 1.4 installed; Windows; Unix; Linux (StarLogo does not seem to be compatible with Java 5/1.5 on Solaris)
SWARM	Swarm Development Group	Java	Windows; Linux; Mac OS X

Note

All software in the table is open‐source and free to use. NetLogo is recommended for ecologists as it hosts an advanced suite of tools for life sciences research.

## 3 WHO WOULD USE AN AGENT‐BASED MODEL?

The flexibility of an ABM makes it a diverse tool for research and teaching. Agents and environments can be arbitrary entities with no real‐life characteristics to test the validity of ecological theory outside the natural environment, for example, the landscape of fear theory (Teckentrup et al., 2018), the theory of trophic ecology (Giacomini et al., 2009), or the dynamics of predator–prey systems (Gras et al., 2009). Alternatively, agents and environments can be realistic representations of wildlife to answer targeted research questions or teach on real‐world systems. For example, Florida panther (*Puma concolor couguar*) movement ecology in a disturbed landscape (Cramer & Portier, 2001), elk (*Cervus elaphus*) migration patterns in Yellowstone National Park (Bennett & Tang, 2006), and coyote (*Canis latrans*) population structure (Conner et al., 2008). On a macroscale, landscape processes can be simulated to study dynamic environmental variables on individual agents for example the effect of oil and gas development on species communities in western North America (Copeland et al., 2009) or the effect of landscape management and structure on multispecies diversity (Goss‐Custard & Stillman, 2008; Hovel & Regan, 2008). All said, the scalability of a model gives the tool a broad usership in science with extensive applications. So, who should use an ABM?

### 3.1 Educators

ABMs are valuable for educators as a simulation tool and as a means to improve computer literacy among their students. Expensive trips to the field for examining ecological processes can be replaced or supplemented by simulating ecological phenomena. Moreover, when using ABMs, students actively engage with adjustable parameters and can witness changing effects in real‐time which can facilitate a greater understanding of theoretical concepts. Here, NetLogo comes into its own as it has a graphical user interface that a student can interact with or without needing to change the underlying code. At the same time, it is important to recognize that quantitative methods and computer literacy are core skill sets of modern ecologists in research and opportunities for exposure to these skills in the undergraduate level are limited (Farrell & Carey, 2018; Read et al., 2016). Teaching computer literacy and code language skills to students who have no experience is a challenge in ecological teaching, especially as these are important skills for a graduate research study (Farrell & Carey, 2018). By embedding these concepts in ecological practical classes using an ABM, students are more likely to engage and increase experience and literacy in computer science (Carey & Gougis, 2016).

### 3.2 Supervisors

Experience with quantitative methods is becoming increasingly important within ecology and evolution as technology and high‐frequency data are heavily used in research (Auker & Barthelmess, 2020; Sherin, 2011). Undergraduate and masters level thesis projects are often students' first attempt at completing an end‐to‐end research project, and this experience is viewed as a proxy for future success and entry to graduate‐level research (Narayanan, 1999). Supervisors have an important and often defining role training their students and exposing them to scientific concepts and methods (Cook, 1980). And those supervisors who have experience using ABMs can equip their students with methods for completing an end‐to‐end research project on a variety of topics while also exposing students to quantitative methods for ecology and evolution.

### 3.3 Researchers

Agent‐based models are data‐fed tools that best simulate realistic systems when high‐frequency data sources are model inputs. Technology in ecology and evolution is quickly advancing the field, and new tools are collecting enormous amounts of high‐fidelity data from sensors and tools deployed in the field (Cagnacci et al., 2010; Hampton et al., 2013; Weathers et al., 2013). Due to their versatility, agent‐based models can be incorporated into a plethora of research projects either to generate data or as an ensemble approach with fieldwork or other quantitative methods. Exposure to these methods at the early‐career stage can diversify a scientist's toolkit and increase accessibility to a diverse research portfolio.

## 4 WHY USE AN AGENT‐BASED MODEL?

In this section, we discuss why educators and researchers should apply ABMs in their teaching and training of students as‐well‐as discussing why researchers can benefit from learning this skill regardless of their field of study.

### 4.1 In teaching

Universities are in the process of altering both taught and research‐based programs to adapt to COVID‐19. To effectively practice social distancing, universities are forecasting that blended learning (in‐person and online teaching) will become standard practice in the 2020–2021 academic year. This presents an opportunity to design courses that promote inclusivity and accessibility for those not capable of physical ecological training. ABMs can be incorporated into blended learning for all student levels to advance their understanding of important topics for scientific training (Shiflet & Shiflet, 2014). ABMs can be used in a variety of ways to diversify teaching methods in a blended environment yet few full courses exist and most users of this method are self‐taught (Macal & North, 2013). Despite this, there is a considerable demand for instruction on how to use this tool across disciplines (Macal & North, 2013).

#### 4.1.1 Seeing is believing

Traditionally in university courses, fundamental concepts in ecology are taught through mathematical equations, for example, the theory of territoriality, niche theory, and predator–prey theory (Bobis, Way, Anderson, & Martin, 2015). Students who struggle mathematically may struggle to engage with these concepts and thus experience stunted learning and feelings of anxiety leading to avoidance of quantitative methods in ecology (Bobis et al., 2015). Despite this, mathematics remains fundamental to ecology. The instruction method remains one of the most important factors in student engagement levels, especially for mathematics (Bobis et al., 2015). ABMs allow students to witness mathematics in action and bridge the gap between mathematics and wildlife.

ABMs allow students to view theory in practice using a graphical interface to see how changing parameters affect patterns in simulations (Shiflet & Shiflet, 2014). NetLogo hosts a library of ready‐to‐run models that are free to use which simulate (with adjustable parameters) a range of concepts such as disease transmission, predator–prey dynamics, territoriality, forest canopy development, ecological disturbance, and succession. These models can be used in parallel with traditional lecturing to enhance understanding or as a valid alternative to practical field components of modules. Especially as more teaching takes place online in 2020–2021 and beyond. By pairing the theoretical explanation of ecological concepts with practical classes using ready‐to‐go ABMs, students can engage with concepts and understand the theory dynamically as they view changes within the model environment.

A survey of students at the postgraduate level ran by Barraquand et al. (2014) found that many students viewed ecology teaching as disconnected from mathematics, statistics, and modeling. Rather concepts were explained as a narrative leaving students underprepared to enter postgraduate studies (Ellison & Dennis, 2010). ABMs are a natural starting point for learning modeling as it is rooted in the individual perspective and demonstrates how individual agents interacting with external factors, like their environment, create complex systems. We would argue that this bottom‐up perspective is more natural for people than top‐down representations through abstract mathematics. Some have suggested that to improve mathematical literacy we should integrate mathematics with field‐courses (Gimenez et al., 2012). We would question if this is realistic? The barriers to fieldwork remain a problem but so is the problem of misunderstanding dynamic systems by observing them at one point in time. ABMs allow students to examine systems in a temporally and spatially explicit environment within the classroom. This method removes barriers to communicating equations effectively and introduces students to quantitative methods, programming, and practical statistics as undergraduates.

#### 4.1.2 Transferable skills

Many students are drawn to ecology due to a love of wildlife and because they believe it is not as quantitatively rooted as other sciences, for example, physics (Barraquand et al., 2014). In reality, ecology always has been and is more and more linked with technology and quantitative methods (Hastings et al., 2005). We must begin to review how students receive training so that they are prepared to advance their careers beyond the undergraduate level. This is increasingly relevant for students hoping to pursue academic research as they will be expected to interface with complex technology, advanced statistics, and big data (Hobbs & Ogle, 2011). In any case, learning programming languages, data handling, and experimental design are transferable skills which improve the employability of science graduates. ABMs expose students to these concepts in the context of ecology and evolution as applicable and engaging coursework.

Software, especially NetLogo, uses a very intuitive programming language which is an easy‐to‐use resource for students looking to improve computer literacy or as an introduction to programming (low threshold for entry, no ceiling for complexity). Students must be trained accordingly as programming and quantitative skills are becoming increasingly important in research throughout the literature (Ríos‐Saldaña, Delibes‐Mateos, & Ferreira, 2018). NetLogo is open‐source, and the model library contains multi‐disciplinary (e.g., ecology, evolution, social science, computer science) model examples that range from very simple to highly complex. All of the code for these models is fully annotated and can be examined, manipulated, and extracted into students own creations. The model development process concludes with the running of experiments and data extraction from NetLogo; thus, ABM also forces students to think about experimental design and data handling throughout the model development process. These are transferable skills that can be extrapolated to projects which involve fieldwork. The ability to use quantitative methods in combination with traditional fieldwork is an attractive attribute for candidates in a competitive job market (Shiflet & Shiflet, 2014; Ríos‐Saldaña et al., 2018).

The scalable complexity of NetLogo also allows students who find themselves interested in quantitative ecology to find new and more complex aspects of the field. As research involving ABMs diversifies so does its applications. Working with R (R Core Team, 2020), Python (Van Rossum & Drake, 2009), QGIS (QGIS, 2020), and other programming languages via NetLogo can lead naturally curious students to expand their quantitative interests, skills, and research portfolio through a relatively simple and early introduction to ABMs.

#### 4.1.3 Research autonomy

Students can also use ABMs in their own projects to complete the full research process for original ideas without a field component. Ecotourism research companies such as Operation Wallacea (https://www.opwall.com/) have been important destinations for undergraduate and graduate students to complete exciting projects in exotic locations. However, this avenue is clouded in uncertainty due to COVID‐19 (Galley & Clifton, 2004; Operation Wallacea COVID‐19 statement). It is often not possible and less practical. however for students, to travel to exotic locations to complete fieldwork for their undergraduate or masters thesis if funding is unavailable.

Research has shown that success of natural history documentaries has raised awareness for many exotic species and led to vicarious connections and public engagement on par with targeted conservation campaigns (Fernández‐Bellon & Kane, 2019). An ABM allows students to select any case‐study species regardless of location and formulate hypotheses of their own choosing without a fieldwork component. An ABM also develops students' experimental design and quantitative methodology in a practical, accessible, and collaborative way. ABMs allow a greater number of students to take full ownership of their thesis project from beginning to end and develop a rounded skill set for scientific research.

### 4.2 In research

Ecological understanding is derived from examining subtle processes, patterns, and interactions that occur between species and the environment (Pressey, Cabeza, Watts, Cowling, & Wilson, 2007). ABMs enable ecologists to study behavioral ecology, movement ecology, intraspecific/interspecific interaction ecology, disturbance ecology, and human interaction ecology across a range of environments; for example, agriculture, forestry, urban landscapes, and wilderness at any spatial‐temporal resolution (McLane et al., 2011). Importantly, ABMs move away from the “average‐individual” paradigm which is typical in traditional models and toward an “ecology of individuals” to capture variance at the core of the system (Uchmański & Grimm, 1996).

#### 4.2.1 Ecology of individuals

The inclusion of tunable parameters, agent behavior, environmental characteristics, and processes in a model sets the ABMs apart. Incorporating subtle variables with radiating effects is a feature not seen in traditional models, for example the role of dominance and territoriality in canid species social structure (Pitt et al., 2003) or the role of previous environmental experience in barnacle goose (*Branta leucopsi*) foraging behavior (Kanarek et al., 2008). For this reason, ABMs differ from other modeling, which are typically written as mathematical equations (Evans, 2012; Pickett, Kolasa, Jones, 2007). The Lotka‐Volterra models are an example of such an equation structure where predator‐prey dynamics are captured by a pair of differential equations. This classical approach to modeling is mathematically tractable in that there are general solutions (Kokko, 2009). Such models are deterministic and include no randomness, they assume we are dealing with a population of “average individuals” (Uchmański & Grimm, 1996). The solutions are continuous which is an acceptable simplification but does lead to the “atto‐fox problem” where fractional populations/organisms (0.5 predators) are possible (Mollison, 1991). In systems where cognition and sensory modality play a part in interactions, ABMs can examine the processes that drive these interactions, for example Srinivasan et al. (2010) modeled predator‐prey dynamics between intelligent organisms in unique habitat (orca *Orcinus orca* and dusky dolphins *Lagenorhynchus obscurus* in Kaikoura, New Zealand).

#### 4.2.2 Biological realism

The inclusion of temporal and spatial extent in an ABM makes it an ideal tool for studying important concepts in biology such as behavior, interaction, movement, and adaptation, for example, Malishev et al., 2017. Models that incorporate physiological barriers and restrictions to behavior are key to capturing biological realism in ecological systems (Johnston et al., 2014). Internal state and navigation drive process in the wild but remain understudied and knowledge on these topics is limited (Graf et al., 2007; Tang & Bennett, 2010). Sense within an ABM dictates where agents go and the resources they access thus having consequences at individual, collective, and landscape scales (Graf et al., 2007). Senses are not universal, and different species have advantages and disadvantages in how they sense their environment (Kalmijn, 1988). The five senses and other nonhuman senses such as electro‐magnetic detection (Keeton, 1971), echolocation (Jones & Holderied, 2007), and ultraviolet light detection (Viitala et al., 1995) provide wildlife with the data they need to make decisions. Including these factors is unique to ABMs and can drive understanding of agent interactions within complex systems.

Interaction is a core concept that underlies ecological theory and has practical consequences in the field (Urban, 2011). Species interact extensively with the abiotic, biotic, and human environment (Gilpin, 1973). Intraspecific interactions within a collective and the group dictates fitness and safety for many species, for example, mammal herds and flocking birds (Seppä et al., 2001; Sparkman et al., 2010; Wang et al., 2011). The behaviors and movement patterns exhibited by groups begin at the individual level. However, how these patterns emerge is poorly understood due to their inherent complexity (DeAngelis & Diaz, 2019). ABMs are invaluable for studying how interactions have collective consequences. This can be applied to understanding arbitrary behavior arbitrarily, for example, flocking behavior and the role of leadership in group dynamics as shown in Quera et al., (2010) or for applications to specific species, for example, examining how group foraging can drive spatial segregation as seen in Northern gannets (*Morus bassanus*) (Wakefield et al., 2013). ABMs are a unique resource for modeling interactions as individual variance is captured as is its effect on the population.

When a model event happens, which changes an agent or environmental state, the output can influence the model in sometimes unpredictable ways. The ability for novel system dynamics to emerge naturally through agent action sets ABMs apart from other techniques (Uchmański & Grimm, 1996). How unconventional patterns emerge can be examined under different parameter values such as habitat heterogeneity which affects foraging behavior (Nonaka & Holme, 2007) or via agent decision‐making such as dispersal (Kramer‐Schadt et al., 2004). When novel systems emerge in an ABM, agents can adapt and thus can be observed responding to a changing environment.

Adaption is a central component of natural science that is difficult to model accurately (Holman, Brown, Carter, Harrison, & Rounsevell, 2019). Agents in ecology change their behavior dynamically over time in response to their environment, and these adaptations can often be unpredictable (Alberti, 2009). Adaption is a fundamental process in ABMs that improves their applicability to ecological research. Changes in the environment may cause a shift in individual behavior which can radiate outward to the collective, for example, examining how elk (*C. elaphus*) adjust their movement ecology to a fire‐disturbed landscape (Rupp & Rupp, 2010) or the adaption of honey bee (*Apis mellifera*) colonies to prevent attacks on colonies capable of a successful defense (Johnson & Nieh, 2010).

#### 4.2.3 Intelligent systems

**FIGURE 4:**
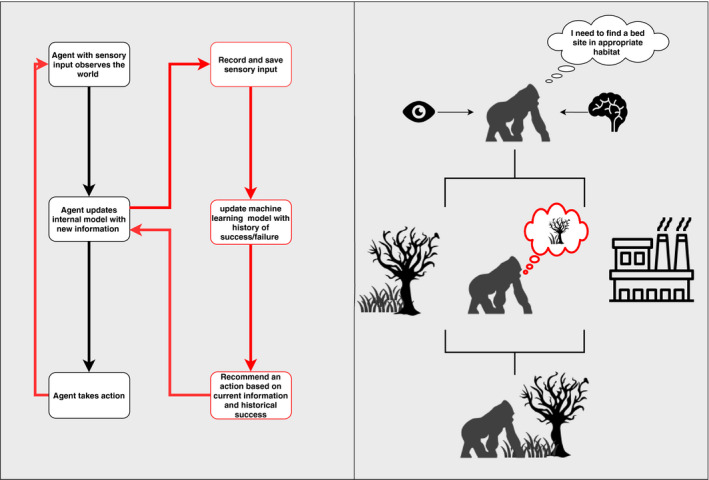
Example of internal agent model cycle (in black) with integration of machine learning (in red) as described by Rand (2006). The left hand side of the diagram shows the logical flow for both a simple internal model (in black) and how that flow changes when machine learning is added for cognition (in red). The right hand side of the model is to visualize how internal models affect behaviour. A simple internal model may tell agents the right choice (in black) whereas via machine learning agents can contextualize the environment and make informed decisions (in red)

Agent decision‐making is a result of internal models which represent cognition. Agent cognition models range from logical if‐then statements to complex algorithms that better mimic animal cognition. Agent learning and decision‐making models have advanced with development in artificial intelligence leading to the integration of these techniques within ABMs (DeAngelis & Diaz, 2019; Rand, 2006). Individual behavior is important in ABMs, and the ability for individuals to develop strategies from experience with a fitness incentive is an invaluable resource for modeling ecological systems (DeAngelis & Diaz, 2019). See Figure 4 for a comparison between a simplistic agent internal model and an agent with integrated machine learning model. Machine learning is a rapidly advancing field and integration with ABMs yields massive potential for forecasting real‐world systems and understanding behavior (Rammer & Seidl, 2019). Using a machine learning model within agents changes the decision‐making process and how they take action over time. Integration of machine learning can also extend knowledge transfer through agent generations and communities which is imperative in modeling species who exhibit complex behaviors. Examples from the literature of ABMs integrated with machine learning algorithms in ecology include neural networks for decision‐making (Okunishi et al., 2009), genetic algorithms for fitness and strategy development (Hamblin, 2012; Mitchell et al., 2012), Q‐learning algorithms for movement (Kons & Santos, 2019), and deep learning for predicting disturbance events (Rammer & Seidl, 2019).

**FIGURE 5:**
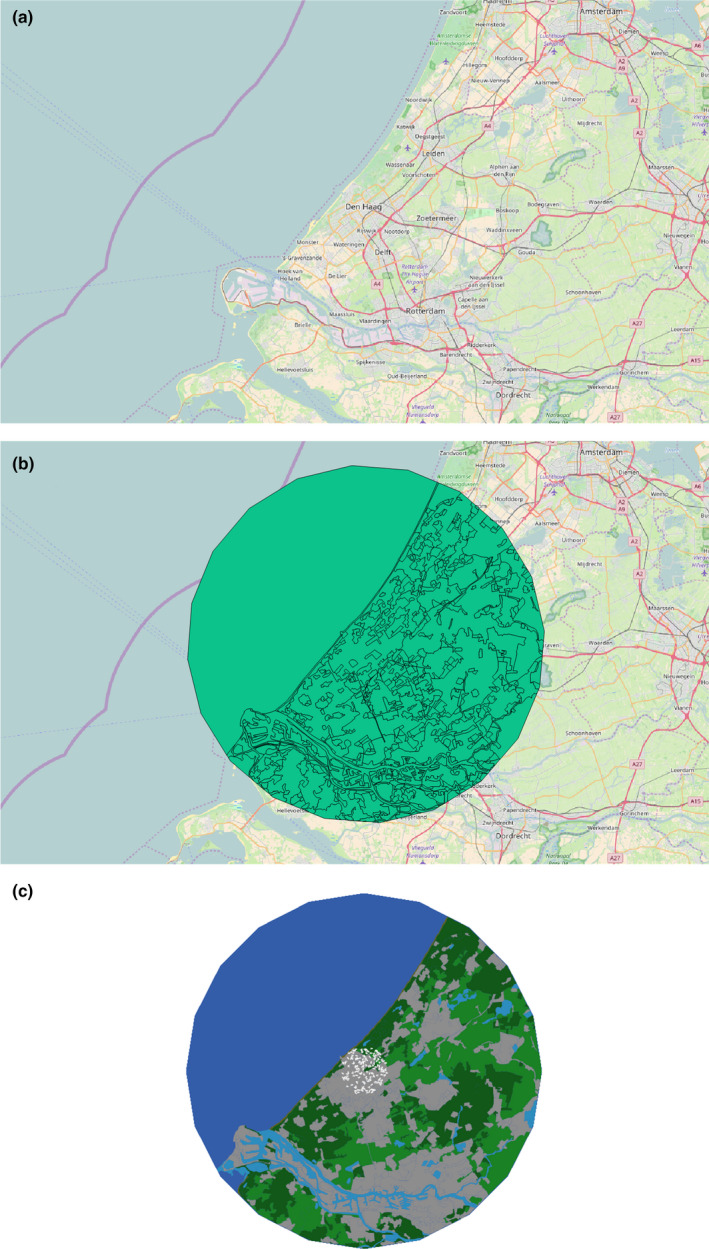
(a) OpenStreetMap view of the city of The Hague, in the Netherlands and its surrounding areas in QGIS. (b) Circle of 30 km radius overlaid with Corine land cover onto map and centred on The Hague in QGIS. (c) View of the NetLogo agent‐based model. The radius of the circle is 30 km. Different Corine land cover types were assigned specific colours, for example, dark blue for sea, light blue for other water bodies, and grey for urban land cover. The agents, in white, represent gulls on their nests and are distributed around the centre of the figure

**FIGURE 6:**
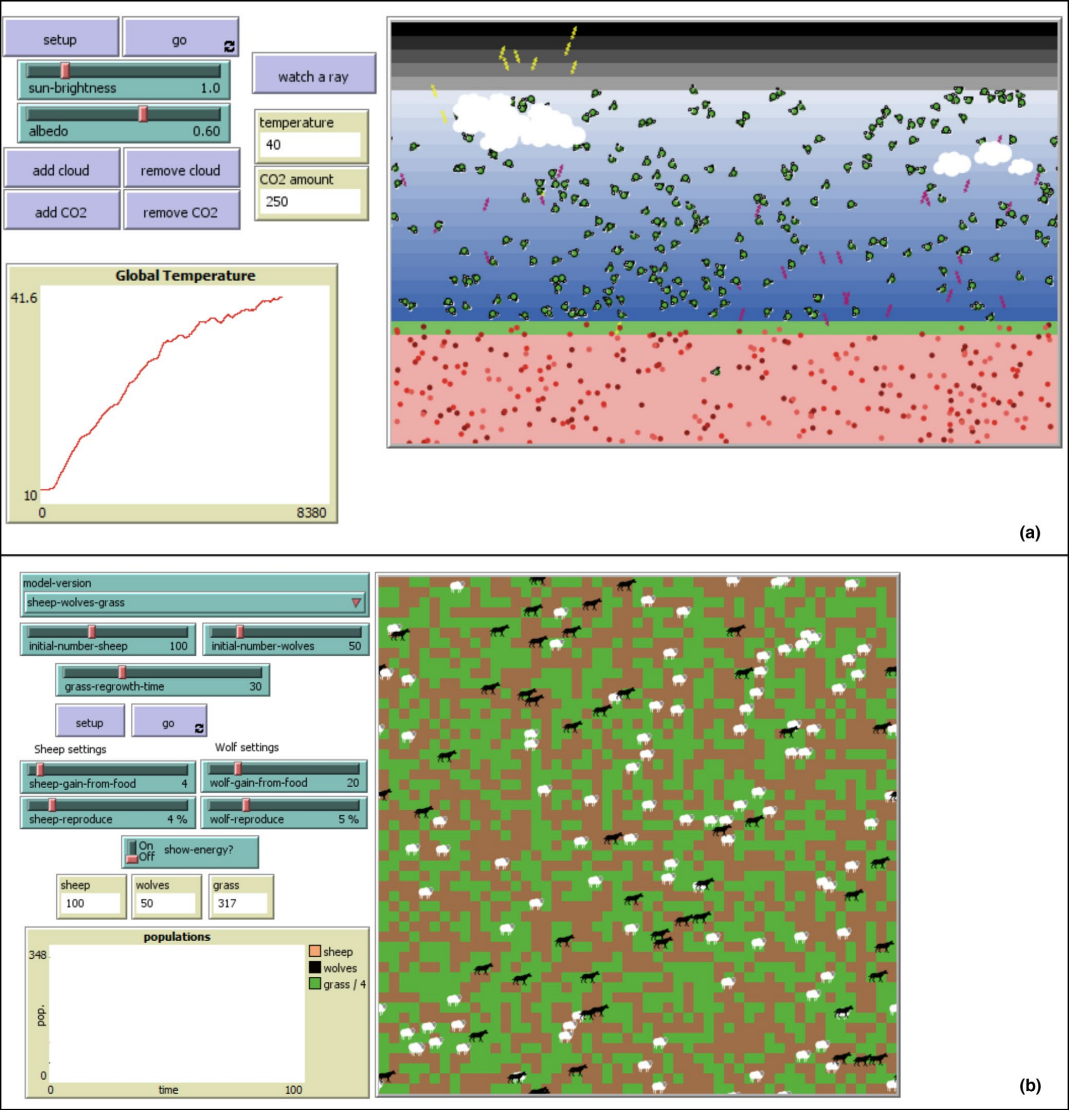
The LevelSpace extension in Netlogo allows two Agent‐Based models to work concurrently. In this example, a climate model (A) dictates the rate of growth for grass, which is a key resource for prey in the predator‐prey model (B). A change in climate parameters in model A will subsequently alter the predator‐dynamic in model B which can affect model A via increased methane release from prey creating a spatiotemporally explicit network of interwoven ecological processes not achievable in most modeling techniques

NetLogo now features a powerful extension allowing for integration of raster and vector shapefiles from GIS datasets (see Figure 5). This high‐frequency input‐data strongly increases simulation accuracy and moves the patchwork environment typical of ABMs toward realistic landscapes (Walker & Johnson, 2019; Wilensky, 1999). Advances are not only being made in creating complex internal models but also incorporating mathematical models and combining multiple ABMs that influence each other in a series of intrinsic interactions using the LevelSpace extension (see Figure 6). Complex submodels improve accuracy through introduction of complex interactions and parameters which alter processes over time and space. Innovation with input‐data is pushing ABMs toward realism and allowing developers to discover new solutions within a representational environment. This promotes repeatability and open‐science as input‐data files can be included in the model package to ensure transparent and scrupulous model development (Grimm et al., 2010; Walker & Johnson, 2019).

Experiments run in NetLogo output data which are typically analyzed in separate software such as Excel, Python, or R (Thiele & Grimm, 2010). Integration of NetLogo with statistical software Python and R has expanded the ABM toolbox as it combines the strengths of both programs to improve accuracy, validation, and ease‐of‐use (Jaxa‐Rozen & Kwakkel, 2018). Both R and Python are open‐source and have a growing user community; thus, integration will allow for the expansion and development of ABMs as a tool with increased capacity for sub‐modeling, parameter testing, and data analysis (Thiele & Grimm, 2010; Salecker et al., 2019; Thiele, 2014).

## 5 HOW TO USE AN AGENT‐BASED MODEL

In this section, we provide learning resources and frameworks for implementing Agent‐Based methods into life science classrooms and research projects.

### 5.1 Tutorials, lesson plans, and hands‐on guidance

We present a range of multimedia learning resources for ABM novices in Supplementary Material 1, which provide fundamental training in the understanding and development of ABM. We provide deliverables and timelines for teaching NetLogo in the classroom in Table 2 to give educators and researchers estimated timeframes for learning the foundation of NetLogo for use in the class and research. We provide a sample lesson plan in Supplementary Material 2 which can be used as a framework for educators who wish to use ABM content to enhance their teaching material. We present links to helpful resources for using ABMs in research in Supplementary Material 3, these resources can equip scientists in the development of complex models to emulate a study site or theoretical hypothesis for peer‐review. Finally, we present a work‐through for a research question in Supplementary Material 4 which active researchers can use as a guide for implementing ABM into their work.

**TABLE 2:** Framework for implementing NetLogo into a teaching schedule

Exercise	Necessary level of understanding	Estimated timeframe
Opening NetLogo and running a ready‐to‐run model	‐ Basic Netlogo functionality (file, edit, tools, zoom, tabs, help; Interface, Info, Code) ‐ Agents (inspect, watch, follow, properties, shape) ‐ Environment (inspect patch, patch coordinates, patch properties) ‐ Global variables ‐ Ticks (tick representation, speed, view updates) ‐ Go ‐ Setup ‐ Plots (viewing)	1–3 hr.
Manipulating a NetLogo model	‐ Buttons ‐ Sliders ‐ Switch ‐ Chooser ‐ Input ‐ Monitor ‐ Plots (designing) ‐ Outputs ‐ Notes ‐ Command Centre	4–6 hr
Basic NetLogo programming *Please see attached simple‐ocean model file for example of basic programming*	‐ Globals ‐ Breeds ‐ Basic procedures ‐ Setup commands ‐ Go commands	6–12 hr
Simple model development for answering basic questions *Please see attached multi‐agent‐ocean model file for example of basic model capable of answering questions*	‐ Creating breeds ‐ Inputting variables (global, patch, turtle) ‐ Creating tunable parameters ‐ Writing functioning procedures ‐ Writing functioning Setup commands ‐ Writing functioning Go commands ‐ Designing experiments in BehaviorSpace	12–30 hr
Complex model development for peer‐review research	‐ NetLogo extensions ‐ Integration of mathematics and code ‐ Understanding spatial and temporal extent ‐ Sensitivity analysis for parameters ‐ Implementing agent cognition and behavior ‐ AOB	30+ hr

Note

Each proceeding exercise assumes understanding of the previous exercise for completion. Two sample models are attached as a framework for what is achievable within the time constraints of the estimated time frames. It is appropriate to quote Hofstadter's law when attempting to estimate time frames, especially with regard to programming; “It always takes longer than you expect, even when you take into account Hofstadter's Law” (Hofstadter, 1999).

### 5.2 To supplement or surrogate fieldwork

ABMs can bridge fieldwork and quantitative methods and allow scientists across sectors to work together (Axelrod, 2006). ABMs can be completely independent of field‐studies and can test theories on abstract or real‐world entities where fieldwork is impossible such as testing the role of body size on obligate scavenging behavior in theropod dinosaurs (Kane et al., 2016).

If fieldwork is central to the project, ABMs can be integrated into the experimental design of field‐based research to generate new insights impossible to collect in the field (Poisot et al., 2019). For example, Carter et al. (2015) used ABMs to model Tiger (*Panthera tigris*) territoriality and population dynamics in Nepal's Chitwan National Park. The ABM prediction accuracy was tested against a twenty‐year field study on tigers in the national park with high accuracy and was used to inform management strategies. Bonnell, Sengupta, Chapman, and Goldberg (2010) showed these techniques in their paper on disease dynamics in red colobus monkeys (*Procolobus* spp.). Dispersal patterns of simulated agents were modeled against field observations with statistical tests to validate predictions on disease dynamics, and they found high accuracy in simulations of real‐world ecological situations which boosted their productivity in the field as they could target disease “hot‐spots” for observation.

Where data exist but fieldwork is not feasible an ABM can be used to save time and resources to test strategies before they are applied in the field. For example, Philips (2020) modeled avenues of dispersal and potential human interactions of Eurasian lynx (*Lynx lynx*) at proposed reintroduction sites in Scottish National Parks to inform spatio–temporal understanding of lynx ecology in an unknown and novel environment.

## 6 LIMITATIONS OF AGENT‐BASED MODELS

Despite the benefits listed above, it remains a challenge to create an ABM capable of generating new insights into ecological systems. While the complex and dynamic nature of ABMs is what makes them attractive, it is also one of the challenges they face. Developing a model system requires the developers to remain focused on the question posed so each facet of the system has the appropriate level of detail for their research objective (Couclelis, 2002; Crooks et al., 2008).

As Orzack (2012) notes “…it is not credible (much less feasible) that we would create a model of ecosystem dynamics that was explicitly grounded in the metabolic dynamics of the cell.” This problem can also extend into the theoretical framework for model development where ad hoc programming can mask important assumptions made in the development, which can skew the outcome of the model (Crooks et al., 2008). All models are simplified representations of reality. This broad definition speaks to the diversity of modeling approaches. Why the need for simplicity? For starters, we are ignorant of the way the world works. We do not have a complete understanding of any process even if we are near certain of the general outline (Breckling, 1992). In ABMs, this is a problem when designing agents and the environment as simplified systems may not accurately model reality.

Certainly, the most potent challenge of ABMs is model validation and calibration. Although leaps in computational power mean researchers can now conduct a comprehensive sensitivity analysis, if real‐world data are unavailable, then validating the results of the model can be challenging and can devalue predictions. There are two solutions to these challenges. Firstly, the ODD protocol was designed to communicate each aspect of model development to reduce ad hoc programming and encourage the developer to justify each model feature with data or references (Grimm & Railsback, 2006). Secondly, by working with field ecologists and building models in tandem with field‐based projects, the outcomes of that model can be calibrated and validated by real‐world data from the system the model is trying to emulate. Despite these challenges, the growth and diversification of the ABMs community birth new ways of thinking and practices to increase the applicability of this tool.

## 7 CONCLUSIONS

In this paper, we discussed the broad applications of ABMs in ecological research. We present the diverse use of this tool in research and teaching across natural science. ABMs can teach students key concepts in ecology and evolution which add to their skill set by introducing them to quantitative methods and tools. The goal of this paper is to advertise ABMs as a resource that ecologists can use to enhance their research, diversify their skillset, and expand their teaching practices. During the COVID‐19 pandemic and looking forward to future unforeseen barriers to fieldwork, ABMs offer alternatives that field ecologists may use. We highlight how the method is continually developing and relies on the growth of a diverse community to offer innovative ways to use this tool and integrate it with other tools of research. Online, open‐source resources present the opportunity to grow professionally during the COVID‐19 pandemic and future disruption of fieldwork.

## CONFLICT OF INTEREST

The authors declare no conflict of interest relating to this manuscript.

## AUTHOR CONTRIBUTIONS

**Kilian J. Murphy:** Conceptualization (lead); investigation (lead); methodology (lead); visualization (lead); writing – original draft (lead); writing – review and editing (equal). **Simone Ciuti**; project administration (equal); supervision (supporting); writing – review and editing (equal). **Adam Kane:** Conceptualization (supporting); supervision (lead); visualization (equal); writing – review and editing (equal).

## Supporting information

Supplementary Material

Supplementary Material

Supplementary Material

Supplementary Material

Supplementary Material

## Data Availability

The authors have no data to present in this manuscript.
