# Supplementary material for: An introduction to agent‐based models as an accessible surrogate to field‐based research and teaching
Source: Ecol Evol. 2020 Oct 2;10(22):12482–98. doi: 10.1002/ece3.6848 (PMC7679541; doi:10.1002/ece3.6848)
Supplement: Supplementary file 2 — Supplementary Material [file ECE3-10-12482-s002.pdf]

Teaching plan example
# - emergent behaviour -

Learning outcomes:

1. Recognise that simple local rules can give rise to complex global patterns.
2. Outline the difference between top-down and bottom-up control.
3. Describe biological systems that exhibit emergent behaviour.

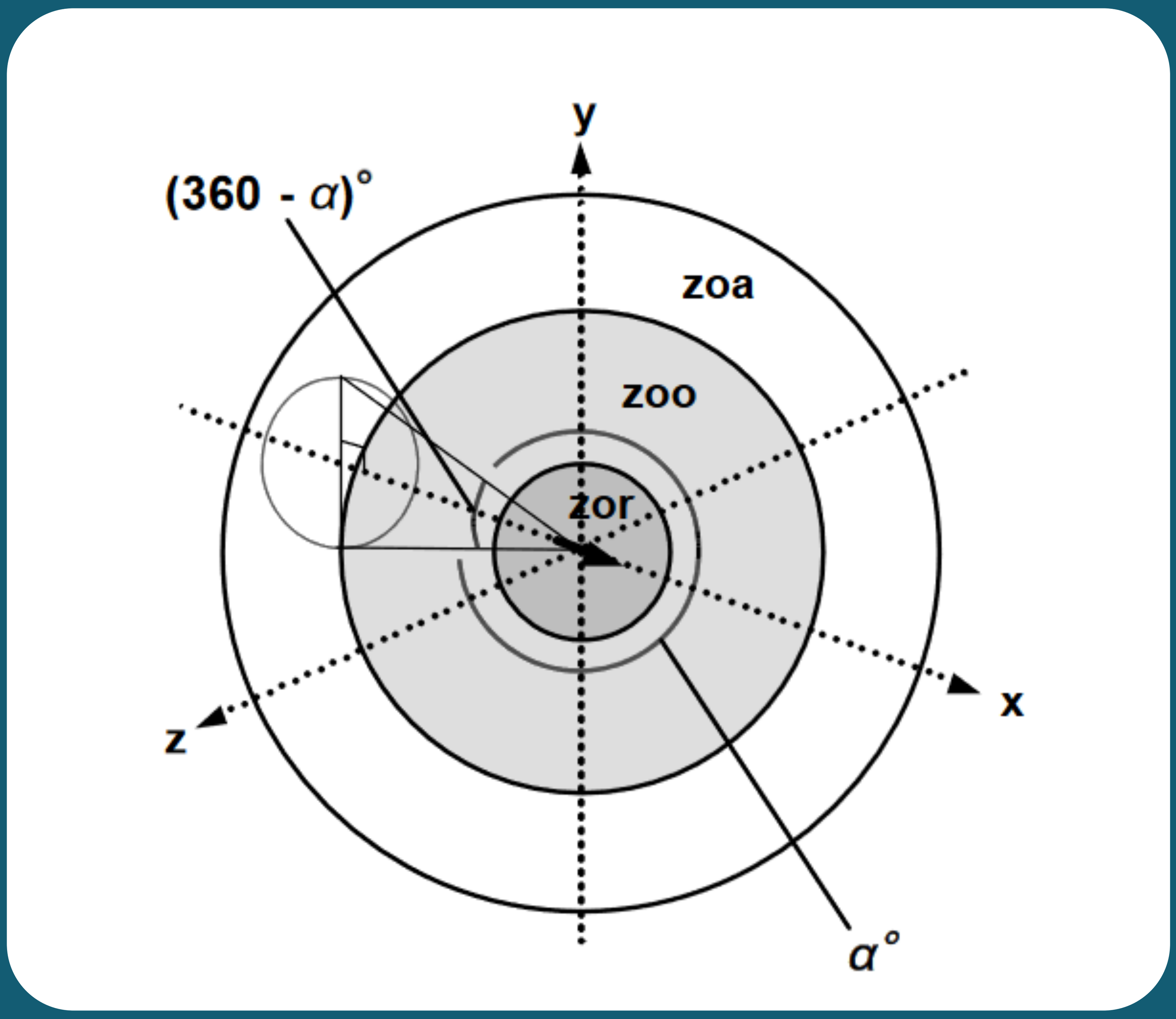

Questions:

1. What are some examples of group behaviour?
2. Is a leader necessary to cause these patterns?
3. What is their biological significance?

Lecture content:

1. Describe emergent behaviour - many definitions!
2. Provide examples from biology e.g. swarming insects, flocking birds and shoaling fish. Starling murmurations provide a great visual image.
3. Explain figure 1 from Couzin et al. (2002) which gives some simple rules that give rise to group behaviour.

NetLogo demo:

Demo the NetLogo model based on the rules described by Couzin et al. (2002) available at: http://ccl.northwestern.edu/netlogo/models/community/couzin

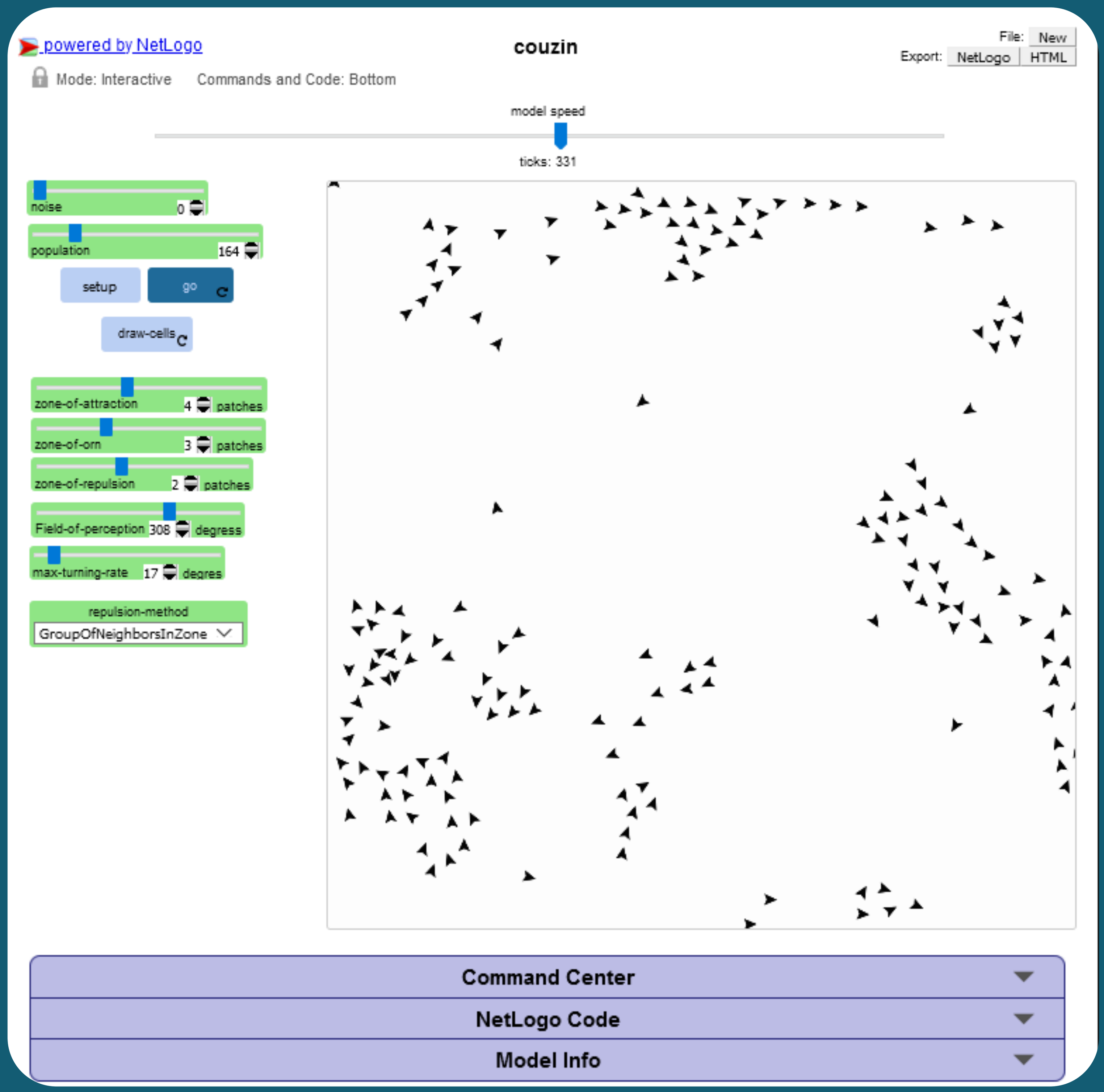

Student engagement:

1. Allow time to understand what each input does by changing the values.
2. Can they 'break' the flocking? What did they do to make this happen?
3. Return to the questions.

Reading:

Couzin, I. D., Krause, J., James, R., Ruxton, G. D., & Franks, N. R. (2002). Collective memory and spatial sorting in animal groups. Journal of theoretical biology, 218(1), 1-12.
