# Supplementary material for: An introduction to agent‐based models as an accessible surrogate to field‐based research and teaching
Source: Ecol Evol. 2020 Oct 2;10(22):12482–98. doi: 10.1002/ece3.6848 (PMC7679541; doi:10.1002/ece3.6848)
Supplement: Supplementary file 4 — Supplementary Material [file ECE3-10-12482-s004.pdf]

Research plan example

# - vulture foraging -

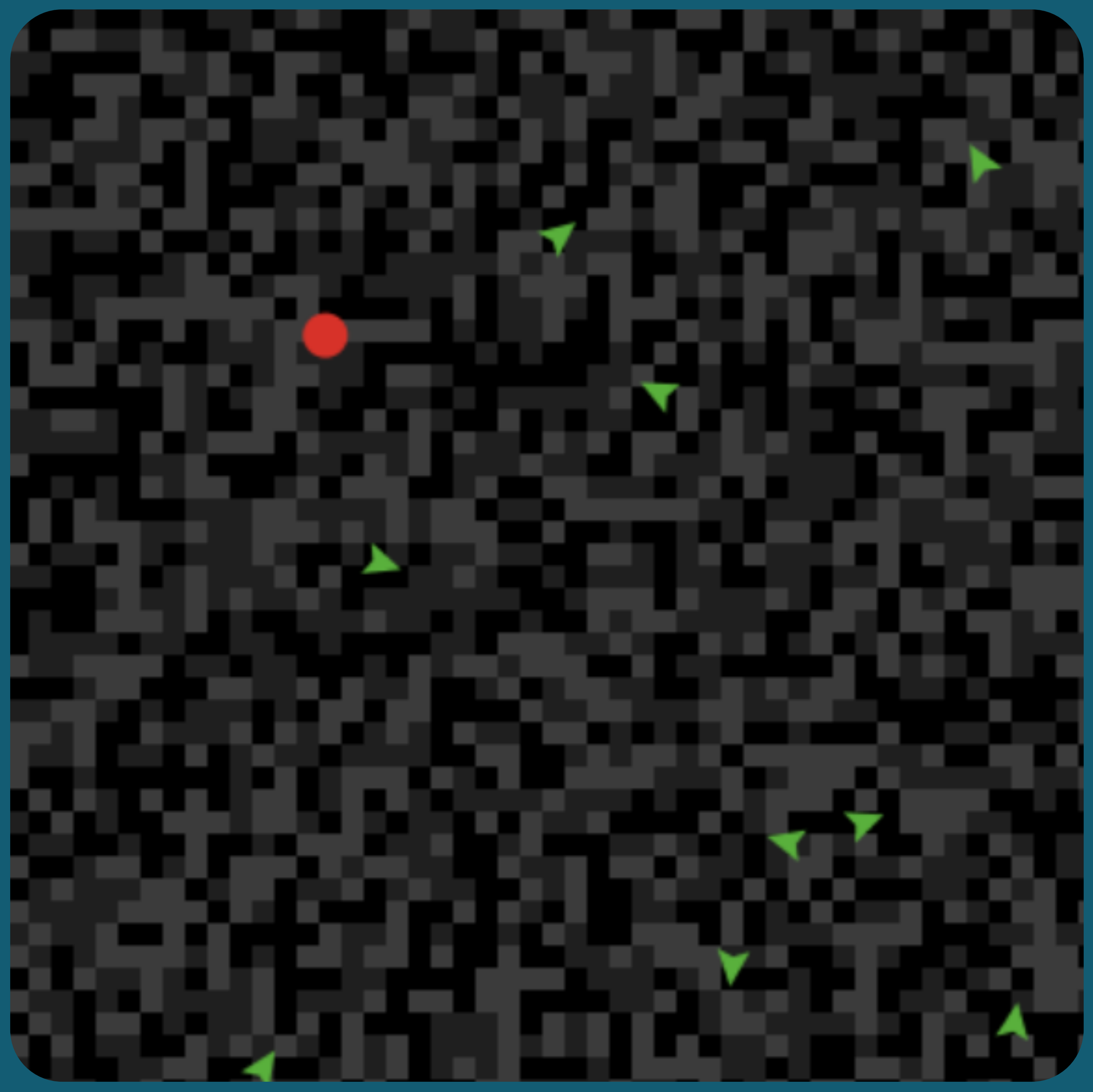

Identify the research question:

Jackson et al. asked how does social facilitation affect foraging success in vultures?

Set out hypotheses

Foraging efficiency will increase with higher visual acuity.
Foraging efficiency will increase with higher carcass density.
Foraging efficiency will increase with higher vulture density.

Structure & code model

Pseudocode and sketches help clarify what you want model to do. Jackson et al. wanted a model of vultures searching for carrion in a spatial environment that mirrored a real ecosystem. Keep parsimony in mind e.g. competition was omitted. Jackson et al. coded it like so:

- Spatial scale: 50 x 50 km, each square = 1 km^2.
- Temporal scale: 1 second model time = 1 second real time.
- Entities: vultures, carcasses.
- State variables: vulture detection distance, behavioural state.
- Processes: searching alone, following another bird, feeding.

```
; entities
breed [carcasses carcass]
breed [vultures vulture]

; state variables
vultures-own [state mycarcass myleader]
carcasses-own [ occupied? ]

; procedure
to setup
  clear-all
  create-carcasses N-carcasses
    [ setxy random-xcor random-ycor
      set color red
      set occupied? "no"
  set shape "circle"]
  create-vultures N-vultures
    [ setxy random-xcor random-ycor
      set color green
      set size 2
      set state "searching"]
  reset-ticks
end
```

Run model and collect data

NetLogo's BehaviorSpace allows user to run model experiments by varying parameters. Jackson et al. recorded time for each bird to find carrion along with values for model parameters e.g. N-vultures, N-carcasses, detection distance.

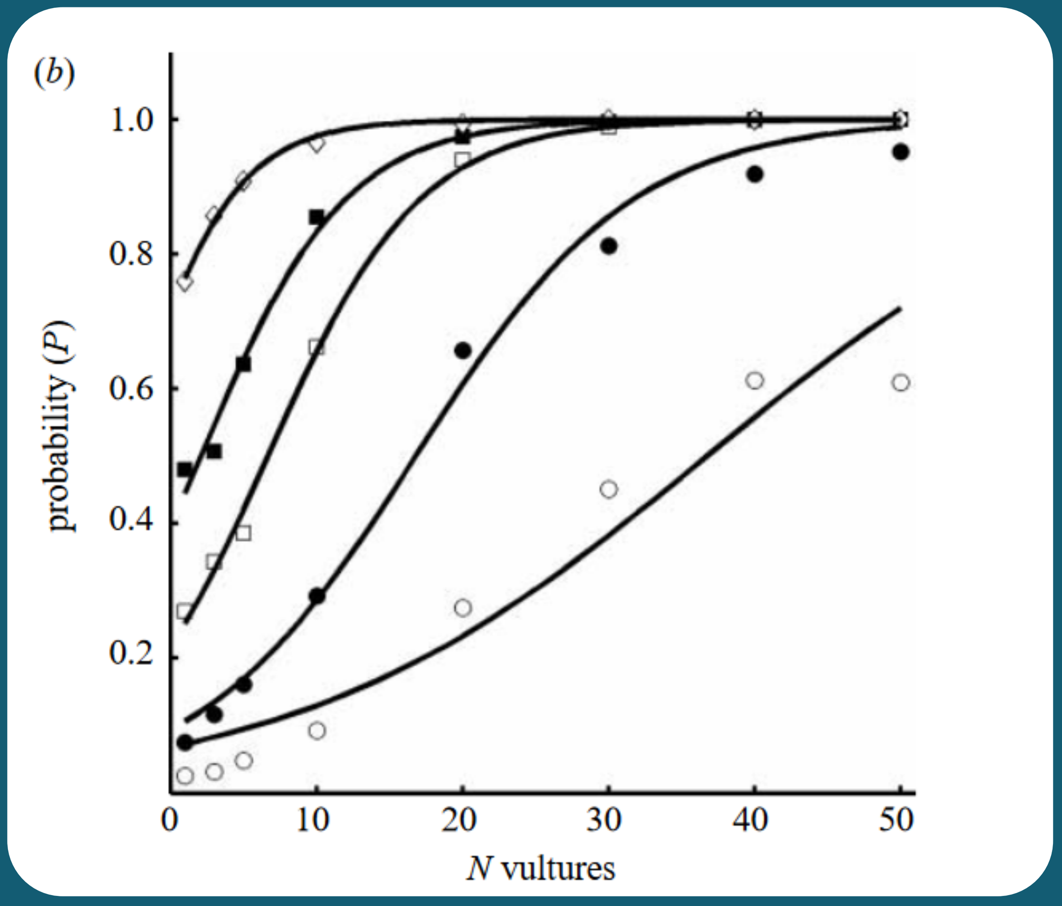

Communicate model & results

Verbally, graphically or statistically communicate findings.
- "Once vulture populations fall below a critical level, the feeding efficiency of each remaining individual falls dramatically...".
Use overview, design concepts and details protocol to explain the model.

References:

- Jackson, A. L., Ruxton, G. D., & Houston, D. C. (2008).
The effect of social facilitation on foraging success in vultures: a modelling study. Biology Letters, 4(3), 311-313.
- Railsback, S. F., & Grimm, V. (2019).
Agent-based and individual-based modeling: a practical introduction. Princeton university press.
