# Supplementary material for: An introduction to agent‐based models as an accessible surrogate to field‐based research and teaching
Source: Ecol Evol. 2020 Oct 2;10(22):12482–98. doi: 10.1002/ece3.6848 (PMC7679541; doi:10.1002/ece3.6848)
Supplement: Supplementary file 5 — Supplementary Material [file ECE3-10-12482-s005.docx]

**Supplementary Material 1.** Community resources for learning Agent-Based Models. These multimedia resources provide an introduction for beginners seeking to advance their knowledge and begin to develop their own models. Links last accessed in August, 2020

| **Resource** | **Medium** | **Link** |
| --- | --- | --- |
| Introduction to Agent-Based Modeling: Modeling Natural, Social and Engineered Complex Systems with NetLogo (2015) by Uri Wilensky (author of NetLogo) and Bill Rand | Book | <http://www.intro-to-abm.com/>  <https://www.amazon.com/dp/0262731894/ref=cm_sw_su_dp> |
| [NetLogo: A Modeling Tool](https://payhip.com/b/VhKb) (2016) by J. C. García Vázquez and F. Sancho Caparrini | Book | <https://payhip.com/b/VhKb> |
| [Agent-Based Spatial Simulation with NetLogo (Volume 1)](http://www.amazon.com/Agent-Based-Spatial-Simulation-NetLogo-Volume/dp/1785480553) (2015) by Arnaud Banos, Christoph Lang, and Nicolas Marilleau | Book | <https://www.amazon.com/Agent-Based-Spatial-Simulation-NetLogo-Volume/dp/1785480553> |
| [Agent-based and Individual-based Modeling: A Practical Introduction](http://www.railsback-grimm-abm-book.com/) (2011) by Steven F. Railsback and Volker Grimm | Book | <http://www.railsback-grimm-abm-book.com/> |
| [Fundamentals of Multiagent Systems with NetLogo Examples](http://www.scribd.com/doc/2094479/Fundamentals-of-Multiagent-Systems) (2009) by José M. Vidal | Book | <https://www.scribd.com/document/2094479/Fundamentals-of-Multiagent-Systems> |
| Netlogo programming language dictionary | Online Dictionary | <https://ccl.northwestern.edu/netlogo/docs/dictionary.html> |
| Agent-Based Modelling for the Self Learner by Jen Badham (June 2019) | Online Tutorial | <http://research.criticalconnections.com.au/ABMBook/> |
| Agent-Based Modeling by Bill Rand (2018) | Youtube playlist | <https://www.youtube.com/watch?v=Z8Wf1vF_xgQ&list=PLF0b3ThojznRKYcrw8moYMUUJK2Ra8Hwl&index=2> |
| NetLogo Community Models | Ready-to-Run models with accessible code | <https://ccl.northwestern.edu/netlogo/models/community/> |
| Netlogo Modelling Commons | Ready-to-Run models with accessible code | <http://modelingcommons.org/account/login> |
| StackOverflow | Community Q&A | <https://stackoverflow.com/search?q=%23netlogo> |
| Netlogo Educators Group | Community Q&A | <http://groups.yahoo.com/group/netlogo-educators/join/> |
| ABM Classroom resources | Resource hub | <http://ccl.northwestern.edu/education.shtml> |

**Supplementary Material 3.** Community resources for using Agent-Based Models in research. These multimedia resources provide resources for beginners to advanced users seeking to increase their knowledge and develop complex models of scientific publication quality. Links last accessed in August, 2020

| **Resource** | **Medium** | **Link** |
| --- | --- | --- |
| [Spatial Simulation: Exploring Pattern and Process](http://patternandprocess.org/) (2014) by David O'Sullivan and George Perry | Book | <http://patternandprocess.org/> |
| [Artificial Intelligence—Agents and Environments](http://bookboon.com/us/textbooks/it/artificial-intelligence-agents-and-environments) (2010) by William John Teahan | Book | <https://bookboon.com/us/textbooks/it/artificial-intelligence-agents-and-environments> |
| Artificial Intelligence – Agent Behaviour (2012)  by [William John Teahan](https://bookboon.com/en/author/0908031c-ce02-9b86-11e6-6dd7d82299d1) | Book | <https://bookboon.com/en/artificial-intelligence-agent-behaviour-i-ebook> |
| Publications which cite NetLogo | Peer-Reviewed papers | <https://ccl.northwestern.edu/netlogo/references.shtml> |
| Agent-Based Modeling by Bill Rand (2018)  Includes modules on GIS, Machine Learning, LevelSpace, etc | Youtube playlist | <https://www.youtube.com/watch?v=Z8Wf1vF_xgQ&list=PLF0b3ThojznRKYcrw8moYMUUJK2Ra8Hwl&index=2> |
| NetLogo Community Models | Ready-to-Run models with accessible code | <https://ccl.northwestern.edu/netlogo/models/community/> |
| Netlogo Modelling Commons | Ready-to-Run models with accessible code | <http://modelingcommons.org/account/login> |
| StackOverflow | Community Q&A | <https://stackoverflow.com/search?q=%23netlogo> |
| Netlogo Extensions (e.g., R, Python, GIS, LevelSpace) | Software | <https://github.com/NetLogo/NetLogo/wiki/Extensions> |
| Running experiments and parameter tuning with BehaviourSpace in NetLogo | Youtube tutorial | <https://www.youtube.com/watch?v=kaOBm6kvEBg> |
| Running a NetLogo model in RStudio | Youtube tutorial | <https://www.youtube.com/watch?v=3EmHi0roiM8&t=535s> |
| Reading GIS datasets into NetLogo | Youtube webinar | <https://www.youtube.com/watch?v=7CAzJjYYtlE> |
